# Empowering rural society through non-formal environmental education: An empirical study of environment and forest development community projects in Ethiopia

**DOI:** 10.1016/j.heliyon.2022.e09127

**Published:** 2022-03-21

**Authors:** Mekonnen Hailemariam Zikargae, Amanuel Gebru Woldearegay, Terje Skjerdal

**Affiliations:** a Bahir Dar University, Ethiopia; b Addis Ababa University, Ethiopia; c NLA University College, Norway

**Keywords:** Environmental education, Stakeholders, Community projects, Ethiopia

## Abstract

This study examined non-formal Environmental Education (EE) is employed in interventions aimed at empowering rural society in Ethiopia. The study focused on a relatively less explored area of how non-formal EE in the form of project-based learning and how it was used to equip the community with skills and knowledge. Using qualitative data collection methods and thematic analysis was employed as an analytical strategy, the study produced evidence of the importance of community programs to out-of-school youths and adults in the acquisition of fundamental skills and knowledge. Greening campaigns were found to be essential in sustaining the lives of the rural communities. As demonstrated in the study, community projects aimed at enhancing learning are in sync with theory of project-based learning. Assisted by relevant conceptualization, the organization which motivated this study, develops insights for knowledge management for further implementation strategies. Its major focus is on empowering poor communities and their institutions by ensuring environmental security and livelihoods. As crucial stakeholders, communities received knowledge and technical skills through experience sharing, training, and workshops. Findings further indicated that most of the project community members were illiterate prior to their engagement in the project activities, which demonstrated that they needed to acquire basic knowledge and skills enact agency. Many community members became part of the project because of the mobilization and awareness creation campaign by the local development organization. However, there have been several roadblocks to the implementation of the community projects. But most importantly the study shows, skills and knowledge imparted through EE were important to implement community projects, helping to enhance community participation in raising environmental quality, thereby improving environmental performance, farming methods, and livelihood situations. We suggest project-based learning be used as a tool for community empowerment initiatives aimed at responding to environmental problems.

## 1 Introduction

Maintaining and enhancing environmental security and the quality of life are continued to be major challenges of our time. The unsustainable utilization of natural resources is a growing concern as climate change and human beings have affected the ecosystem balance and biodiversity (Umoh, 2010; Dou et al., 2020, 2021). Biodiversity loss threatens human wellbeing and vice versa (Diaz et al., 2006; Pires et al., 2020). The natural link between biodiversity and human wellbeing ought to lead society towards sustainable development (SD) (Naeem et al., 2016). In discourses of the environment, Ethiopia can be taken as an important example of the relevant perils and promises (Kebbede, 2016; Kassa et al., 2017). Although the country is blessed with natural resources, the possible benefits for its people have been limited due to its unsustainable management and utilization of its natural resources, which have led to a gradual decline in agricultural production and productivity (Birhanu, 2014; Daley, 2015; Wassie, 2020; Zikargae et al., 2021; Zikargae, 2018, 2021).

Besides, the depletion of natural resources, low use of direct inputs, and dependence on rainfall for the agricultural sector as well as limited use of practice of innovative technologies and practices have contributed to reduced productivity (Tesso, 2019; Gashaw et al., 2014; Villena and Greve, 2018; Daley, 2015; Adegbeye et al., 2020). To this end, studies indicate that environmental challenges and climate change problems constitute deep-rooted economic, social, and environmental obstacles in Ethiopia (Gashaw et al., 2014; Zikargae, 2021; Wassie, 2020; Daley, 2015). Dependence on rain-fed agriculture, adverse economic development, deforestation, land degradation, and larger as well as denser human settlements are alarmingly increasing human and environmental vulnerability in the country. The problem has captured both global and local attention.

The Amhara region is one of the distinct regions with the most widespread and chronic food insecurity, environmental and sustainable challenges in Ethiopia. This is partly because the region is mountainous and densely populated. Besides, the area is located in the Ethiopian highlands. Due to mountainous nature, regional farmers have limited arable land. Production means have been unsupported by technology. So it has been limited to subsistence farming for centuries. To overcome the challenges emanating from unwise relationships between nature and human beings, several actors have developed global instruments for community interventions in rural areas (Zikargae et al., 2021; Zikargae, 2021; Zikargae, 2020; Ali and Sonderling, 2017; Godana, 2014).

The contribution of non-governmental organizations (NGO) is one step in supporting the government's commitment to the improvement of environmental security, forest development, SD challenges, and fighting climate change (Zikargae et al., 2021; Zikargae, 2021; Zikargae, 2020; Godana, 2014). The Organization for Rehabilitation and Development in Amhara (ORDA) is intensively working in the Amhara region to fill in the government's gaps regarding environmental and sustainable livelihoods challenges. However, studies show that the Ethiopian government lacks coordination and effective communication with different stakeholders, local development organizations and environmental promoters (Horwood, 2020; Mulugeta et al., 2019; Zikargae, 2018; Zikargae and Ali, 2017; MoEDC, 1997). This study looks at the sustainable practices of ORDA's community interventions using non-formal EE by increasing information exchange, environmental awareness and skills (Zikargae, 2021) of the targeted communities' projects to combat and fill gaps in environmental challenges and climate change.

This study focuses on non-formal EE as a component of participatory environmental communication (PEC) used as an instrument to recognize the process of environment and forest community interventions implemented by ORDA in the Amhara region. The nonprofit organization offers a tremendous experience of implementation of community projects for rural communities. Thus, it is considered ORDA's practical implementation of the Environment and Forest Development Program (EFDP). Studies confirm how non-formal EE is managed as a tool to combat various environment and development-related opportunities and challenges (Yeshalem, 2013) of the rural community.

Above all, the current study focuses on managing non-formal EE to impart behavior, motivations, attitudes, skills, knowledge, and awareness (hereafter B-MASKA) as applied to the poor communities of Enfiranz, Derita, Muja and Eyella. The interventions are aimed at the alleviation of environmental and livelihood challenges through the practice of integrated community participation. Scholars argue that it is necessary to address environmental challenges by enhancing the environmental awareness and literacy of participants (Stern et al., 2014). Capacitating the community and their institutions is a prerequisite to effectively communicating with the stakeholders. The capacity has been regained through project-based non-formal EE. ORDA has, more or less, been using such instruments.

According to the organization's self-presentation, ORDA works to "empower impoverished communities and their local institutions to achieve livelihoods and environmental security in the Amhara region" (2019a, p.i). The communities have been engaged in forest and greening programs in the effort to sustain livelihoods. However, they continue to face the challenges of dealing with very little arable land, changing climatic conditions, watersheds, and rainfall flooding problems.

Given the socio-economic backgrounds, it is challenging for individuals and communities to make ends meet. A way out will require increased afforestation, greenness and improved land conditions, thereby improving the environmental security and livelihoods of the community. Providing and sustaining ecosystem services to project communities are mandatory for the survival of the community. The evidence shows how non-formal education programs are being used to eradicate abject poverty by imparting knowledge and skills (Teane, 2021). This study intends to understand how the lives of communities have been improved through the use of non-formal EE as key strategies to community project. The innovative strategies involve project-based learning, with community members coming together, producing, sharing and exchanging creative ideas. The activities of non-formal PEC help the community to address various activities. Unless the specific activities are considered PEC are implemented, the community project is unable to adequately meet the desired outcome.

These activities ensure PEC at the grassroots level rather than a top-down approach. The capacity building of communities and their institutions plays a vital role in enhancing mutual learning and experience sharing (ORDA, 2019b). The possible use of community interventions is vital as a specific form of environmental activity through experiential learning. This involvement enlightens community members about successful strategies to respond to environmental challenges. Scholars have indicated how using project-based learning facilitates and promotes community engagement to standard practice (Mead et al., 2012). Relevant academic research has been conducted globally, but studies focusing specifically on Ethiopia are insignificant.

The objectives of this study are twofold: First, it aims to analyze how ORDA considers non-formal EE as a tool to engage and communicate with its stakeholders; secondly, it seeks to identify the challenges faced by ORDA in applying non-formal EE as part of PEC on community projects. The empirical findings reveal the key skills, favorable attitudes, and practical knowledge (B-MASKA) imparted by the ORDA. The findings can be used to support the community's farming methods, livelihoods, and forest and watershed management. Training has increased crop yield, water retention, soil fertility, mountain landscape, and environmental security. The effective management of B-MASKA affects the engagement implementation of community projects. The Non-formal EE as one of the key components of PEC is overlooked and limited in contemporary academic discourses and research. However, it is considered vital in ORDA's community interventions. This study, therefore, provides important results for EE and the implementation strategies that contribute to the sustainable development of a region.

## 2 The non-formal environmental education

The integrated theory of EE guides the study by combining participatory theory with non-formal EE components and concepts. Non-formal EE components, particularly B-MASKA, are major concepts in participatory theory and have been used as frameworks for the current study.

Two forms of environmental education are highlighted in the ligature: formal education that is used in the curricular and outdoor education, and non-formal education used for implementing community projects. Environmental education is a general form of education used to implement community projects. Across the international system, intergovernmental international treaties have drawn attention to lending credence to its relevance. As has been manifestly made clear, the agreements on EE are aimed at combating environmental and SD challenges (Tbilisi, 1977). Agreements are used as instruments to deal with these challenges. The agreements also provide directions on the modalities of intervention as well as a general framework to improve environmental security in a global context.

In less formal contexts, the relevant, literature shows that non-formal EE is practiced in outdoor and environmental contexts (Zikargae, 2021). It is further taken as an integral part of SD, stakeholders’ participation, and environmental communication (Cox, 2010). The link between EE and SD was emphasized by an EE and SD conference on environmental education and found further traction in subsequent periods (Tbilisi, 1977). The concepts, goals, and approaches gained momentum at the Stockholm UN conference in 1972 (Astrachan, 1972). The conference motivated 175 countries to focus on policies that address environmental challenges and development issues. Countries were expected to include and demonstrate their action-based commitment to environmental issues as a cross-cutting issue in their policies.

The Stockholm conference culminated in a Declaration having principles relating to the environment and development as well as an action plan with a set of recommendations (Baylis and Smith, 2005). A particularly relevant subject of discourse of the conference was the realization of the impact of poverty on the environment as well the need for environmental education and research. These points continue to resonate across the developing world as the importance and fragility of the environment have become more pressing issues. There is more recognition of the role of environmental education, reflecting the need for a more responsible use of the environment.

Premium is placed on environmental literacy as a key input. Obtaining knowledge and skills relating to socio-ecological outcomes of community projects empowers citizens and engages them in a dialogue about local and global environmental and sustainability issues (Dillon, 2018). Environmental education provides information and is an international learning tool that offers a synergistic public space to take action on environmental issues. Further, it is an important tool to implement the community projects and more broadly a global instrument or tool that influences B-MASKA. EE is also a tool used to shape attitudes, and enhance knowledge, and skills to address challenges (Ardoin et al., 2020). It is further argued that EE plays a significant role in poverty reduction in Africa (Umoh, 2010). Thus, EE, as a component of EC, is viewed as an important instrumental in filling the gaps in attitudes, knowledge, and skills required to establish and maintain the environmental security and the dignity of Ethiopia's poor society.

### 2.1 Strategies of environmental education

The EE strategy is a multi-level and grassroots intervention with continuity. The process of EE can occur through non-formal and informal approaches. The non-formal strategy targets special project-based programs outside the school system for adults and communities (Talero, 2004; CEgN, 2006). As part of a life-long mutual learning process, environmental programs are provided for the community, organizations, youth groups, and nature centers. It is an integral component of lifelong learning (Paraskeva-Hadjichambi et al., 2020). These include stakeholders' participation in workshops, volunteering, role plays, field trips, holidays, field visits and environmental days. It is also noted that EE involves the dissemination of information without an organized educational structure. It includes mutual learning about the environment through the media, personal reading, everyday experiences, and interactions with other people, and sharing their experiences. This type of learning entails experiential learning based on community projects. EE goes beyond creating awareness, instilling knowledge and skills (UNCED, 1992). It brings behavioral and attitude change to taking environmental actions.

The literature points out that EE is more than learning about the environment; rather, it is about changing behavior. After changing human behavior, it instills learners' knowledge of the environment to take action. Hence, the community develops positive attitudes towards environmental challenges. Positive attitudes boost the competency of a citizen's acting skills. It could help the community to develop a sense of empowerment and legitimacy (Rennie, 2001; Athman and Monroe, 2001). Empowerment and legitimacy promote and achieve the objectives of the organization.

Some of the objectives of EE are as follows: promoting environmental awareness, behavior, and eventually action through education, and raising stakeholders' awareness and training about their environment (Hens, 1997; CEgN, 2006; Gough and Gough, 2010; Frantz and Mayer, 2014). Here, the integrated-multilevel approach to bringing behavioral change at both the individual and societal levels is important to improve stakeholders’ skills and knowledge to tackle existing and potential environmental challenges. Acquiring knowledge could bring changes in attitudes and behavior.

### 2.2 B-MASKA (behavior, motivations, and attitudes, skills, knowledge, and awareness)

Non-formal EE in the form of B-MASKA development, information exchange, community empowerment, environmental performance, and livelihood improvements, remains an effective tool for addressing environmental and sustainability challenges. For developing and enhancing environmental B-MASKA partnerships, and building skills for engaging communities in collaborative environmental learning and actions, EE represents more than just a one-way information flow (Ardoina, Bowersd & Gaillard, 2019; Paraskeva-Hadjichambi et al., 2020; Tbilisi, 1977; Athman and Monroe, 2001; Cesar and Ekbom, 2013; UNEP, Belgrade Charter, 1975). Further, EE provides a way to discuss global environmental challenges and their consequences, including poverty. Environmental degradation and poverty are said to have a complex relationship (Bekalo and Bangay, 2002). Frequent drought and land degradation are thought to have affected 169 countries, with the poorest rural areas suffering the most (IPS, 2021). The expansion of the rural population, limited land resources, and many people reliant on land resources are said to represent the drivers of such a performance. The Tbilisi Agreement, signed in 1977, established the goal of EE to provide information frameworks at the local and international levels that consider B-MASKA and participation to assist and empower stakeholders in taking practical environmental action (Tbilisi, 1977; Athman and Monroe, 2001; Cesar and Ekbom, 2013). The present drive for comprehensive and holistic EE stems from Agenda 21, which was approved in 1992 at the Rio Conference. Other assertions and official declarations appeared in the Tbilisi Declaration and the Belgrade Charter (UNEP, 1975), both of which include training and public awareness (1977). Planning and conducting an EE program require comprehensive frameworks. Maintaining a healthy receptive-responsive interaction with key components of nature is crucial to human well-being (Bonnett, 2019).

In the Ethiopian society, subsistence and export agriculture are dependent on the land. Drought and resource degradation, on the other hand, have had an impact on land productivity for many years, affecting many agricultural products (Wassie, 2020; Bekalo and Bangay, 2002). Together climate change, soil degradation, deforestation, loss of biodiversity and ecosystem services, and exploitation of natural resources are all identified as environmental issues in Ethiopia (Wassie, 2020; Cesar and Ekbom, 2013; Tbilisi, 1977; Athman and Monroe, 2001).These difficulties result in reduced economic growth, and fewer prospects for a livelihood. The extensive eco-disaster has been described as having the hallmarks of a dystopia in the worst-case scenario, with no signs of improvement. However, there are prospects for reversing environmental degradation and climate change outcomes (Hope, 2007). Among possible strategies for reversing the spiral is relevant awareness development through environmental education.

Environmental awareness creation is a technique for improving environmental B-MASKA as well as providing the experience of changing community beliefs, attitudes, and behaviors (Frantz and Mayer, 2014; Choe et al., 2020). The environmental B-MASKA gaps have persisted in the community, and community projects can provide opportunities for hands-on experience learning about how to deal with environmental concerns. Experiential learning represents a key strategy for acquiring knowledge and skills by doing things (Paraskeva-Hadjichambi et al., 2020; Efstratia, 2014; Falk, 2005) in non-formal EE settings (Ballantyne and Packer, 2005) where community-based organizations take the lead in addressing human-caused environmental problems (Saribas et al., 2017). Evidence demonstrates that non-formal EE can improve environmental quality and output by teaching environmental and watershed management skills and knowledge (Paraskeva-Hadjichambi et al., 2020). One of the goals of EE could be to look at how the community is related to nature as a predictor of environmentally responsible behavior (Frantz and Mayer, 2014). According to Paraskeva-Hadjichambi et al. (2020), such community-based interventions promote social change in a particular community. Much environmental education in Ethiopia is academic in character and discourses on more focused, village/community centered practical interventions are extremely limited (Bekalo and Bangay 2002).

The environmental problems in the Amhara region have gone unnoticed. They have emerged partly as a result of political pressure to address societal difficulties. The issues are exacerbated by a lack of government commitment and funding (Wassie, 2020). However, several local non-governmental organizations (NGOs) attempt to respond to the challenges. The implications of non-formal EE skills, attitudes, and knowledge to improve EFDP in the regional parts of the Enfiranz, Derita, Muja, and Eyella project communities have been identified in this study.

## 3 Method

A qualitative/interpretive research methodology is used to frame the overall research design of the study. The design refers to a process that determines what, who, how to collect and analyze the data (Creswell and Creswell, 2018; Lune and Berg, 2017). The data were interpreted using thematic analysis.

A case study approach is used to circle in on ORDA's activities. The organization is suited for the current study because it has community intervention projects on EFDP. It has had a significant impact on the rural society of the Amhara region's environmental security, livelihoods, and SD. Besides, there are three similar community projects which are included as cases of the study. The rationale for choosing a case study is that it systematically investigates an event or set of events by describing and explaining these phenomena (Lune and Berg, 2017; Yin, 2018). The case study also uses multiple methods to provide a full and deep examination of the case (Creswell and Creswell, 2018). Explanatory and descriptive case study designs are used in the current study (Lune and Berg, 2017).

### 3.1 Population and sampling contexts

The research participants came from Enfiranz, Derita, Muja, and Eyella project communities, representing the Central Gondar, South Gondar, and North Wollo Zones of the Amhara region in the North-Western part of Ethiopia. The research made use of dialogues and observation of participants who were engaged in greening, watershed, and livelihood programs. Ten in-depth interviews with seven project community members and three experts, as well as six focus group discussions with thirty-six project community members were undertaken, allowing the research team gather as much information as possible. Therefore, in the current study, a total of forty-six project community members participated.

The target population comprises all the project communities of Enfiranz, Derita, Muja, and Eyella intervention sites. The population of the study is considered active participants in the community and experts engaged in community projects.Both convenience and purposeful sampling techniques were used in the study.

**Figure 1:**
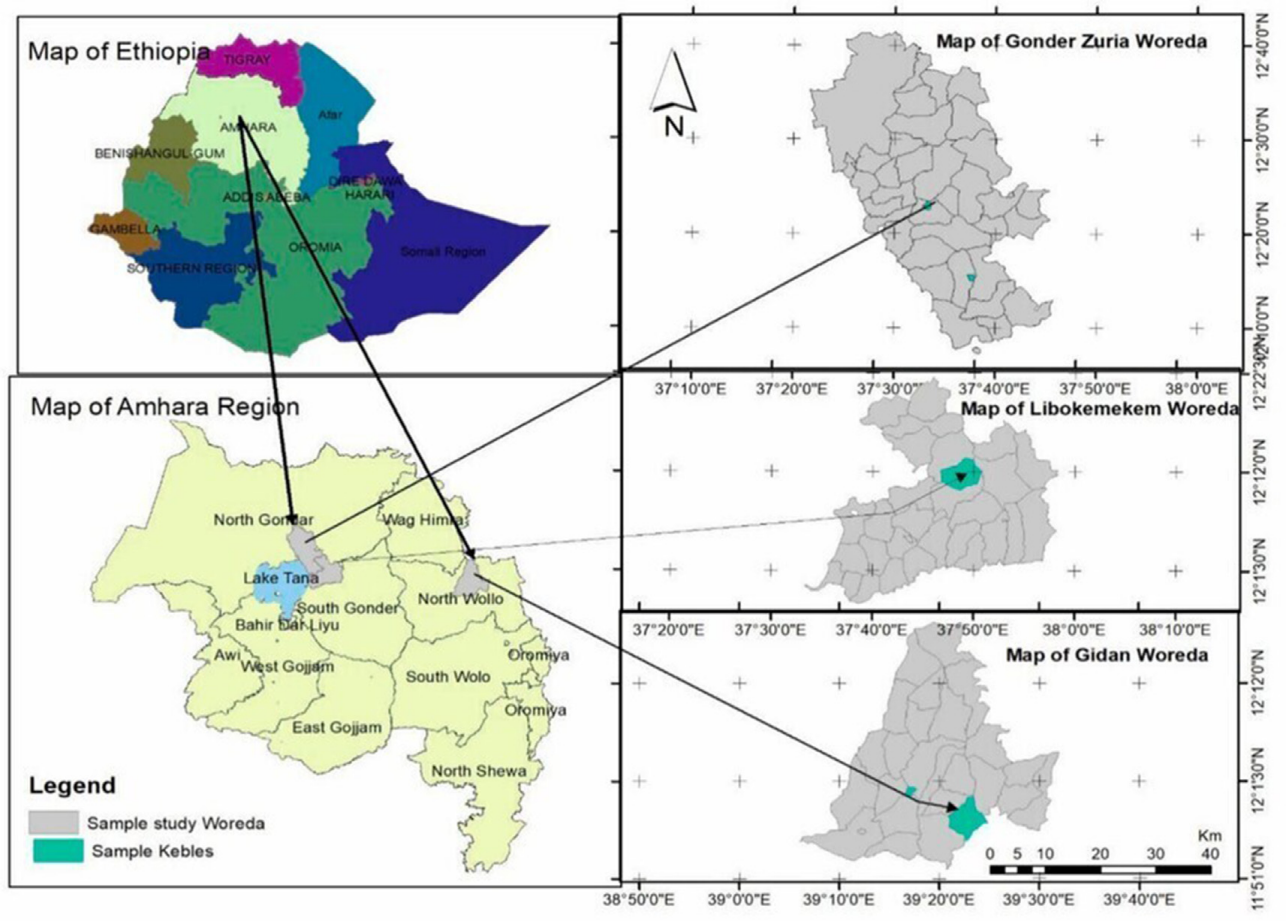
ORDA's intervention sites (Zikargae, Credit: sketched by Fikirte).

The scope of the current study focuses on ORDA's active intervention sites. ORDA has been working on three intervention sites implementing EFDP in three zones: Central Gondar, South Gondar, and North Wollo, which include the Gondar Zuria, Libokemkem, and Gidan districts, respectively (Figure 1). The sites are purposely selected based on the possible duration of the intervention. The interventions have continued since 2000. The zones are described as high mountains and contain communal land that could be available and suitable for EFDP. The interventions have continued for almost two decades in Eyella village, whereas the Muja project was implemented after three productive years of intervention. The rest have followed the Eyella and Muja interventions. The findings of this study show that the Muja project community members have immediate exposure to the Eyella projects. The Muja's intervention project community members communicate since they are close neighbors and have experience of willingly sharing with their neighbors in the Eyella village. The Enferanz and Derita intervention sites have been engaged for a decade. All sites receive learned and shared experience from other areas. Key members of the community have been practicing greening, watershed programs and livelihood enhancements. Several enhancement tools are used to upgrade knowledge and skills. The study obtained their views of the Enfiranz, Derita, Muja, and Eyella participants through dialogues and empirical observation.

Sampling criteria are developed scientifically based on previous theoretical and practical experience (Martinez-Mesa, 2016; Mosera and Korstjensc, 2018). The participants are selected for they were able to share actual experiences; participatory engagement (training, forums, planning, clubs, watershed committee); subject articulation; able to talk about their challenges; shared experiences; volunteer to participate; age/sex; experience/services in the community, roles in the community.These sampling criteria are used to select an essential part of the local population. Except for the leading experts’ proportional representation, the specific individual and group interviews applied similar criteria for the practical purpose of the comprehensive study. Experts were selected based on experiences, expertise and their availability.

The study used two types of empirical material. The first type consists of document analysis of scientific reports, proposals, and strategic plans of ORDA as well as different international agreements and protocols of non-formal EE are as discussed in the ORDA documents that are reviewed. However, the principal activities of data collection began with document reviews. This initial data exploration was made easier because ORDA possesses experience in documenting strategic plans, reports, and project proposals. All the documents describe how non-formal EE has helped to achieve the organizational objectives and explain how it is used to alleviate environmental challenges for the community. The other type of material is empirical data analysis addressed in-depth interviews, focus group discussions and direct observation.

Besides, we conducted six focus group discussions (Table 1) revealed current perspectives on issues around project-based learning the discussions provided more insight into how non-formal education impacted their communication, information exchange, and management of environmental activities as well as security and livelihood improvements. Moreover, we collected empirical data by observing what had been done. We visited all the intervention sites and collected pictures of the project sites where nursery, seedling, greening, and watershed management were underway. Greening and watershed management require nursery and seedling site preparation. In the document analysis part of the study, project proposals, strategic plans, performance reports, and research documents were reviewed. To this end, direct observations were carefully conducted during field visits.

**Table 1:** Data gathering procedures (46 participants).

*Stage 1: Document Analysis*		
Reviewed: Strategic plans, project proposals, annual reports, website contents etc.		
*Stage 2: Project-Community Members*		
In-depth interviews & focus group discussion: 43 participants from Enfiranz, Derita, Muja & Eyella included		
Activities	Seven community members participated in the interviews: CI1,CI2,CI3,CI4,CI5,CI6,CI7	Purposive sampling
Activities	Thirty-six project community members participated in discussions: FGD1,FGD2,FGD3,FGD4,FGD5 & FGD6	Purposive sampling
*Stage 3: Experts in-depth interviews*		
Activities	Three interviewees conducted: EI1,EI2,EI3	Convenience sampling

Following document analysis thematic analysis was undertaken. As used in this study, document analysis/review is a method of study involving the systematic interpretation of relevant documentary evidence to shed light on issues relating to an enquiry (Bowen, 2009).

Primary data analysis used the thematic analysis while thematic analysis is a method that identifies key themes of interest through coding procedures (Lune and Berg, 2017; Creswell and Creswell, 2018). Moreover, thematic coding or analysis of both sources is used to analyze the collected data.

Therefore the analysis section presents results and discusses the data in two complementary phases: document and primary data analysis. Based on thematic analysis, the study is expected to answer two questions:

Q1: How does ORDA consider non-formal EE as a tool to communicate with and engage its stakeholders?
Q2: What are the communication challenges faced by ORDA in applying non-formal EE to EFDP community projects?

## 4 Results and discussion

**Figure 2:**
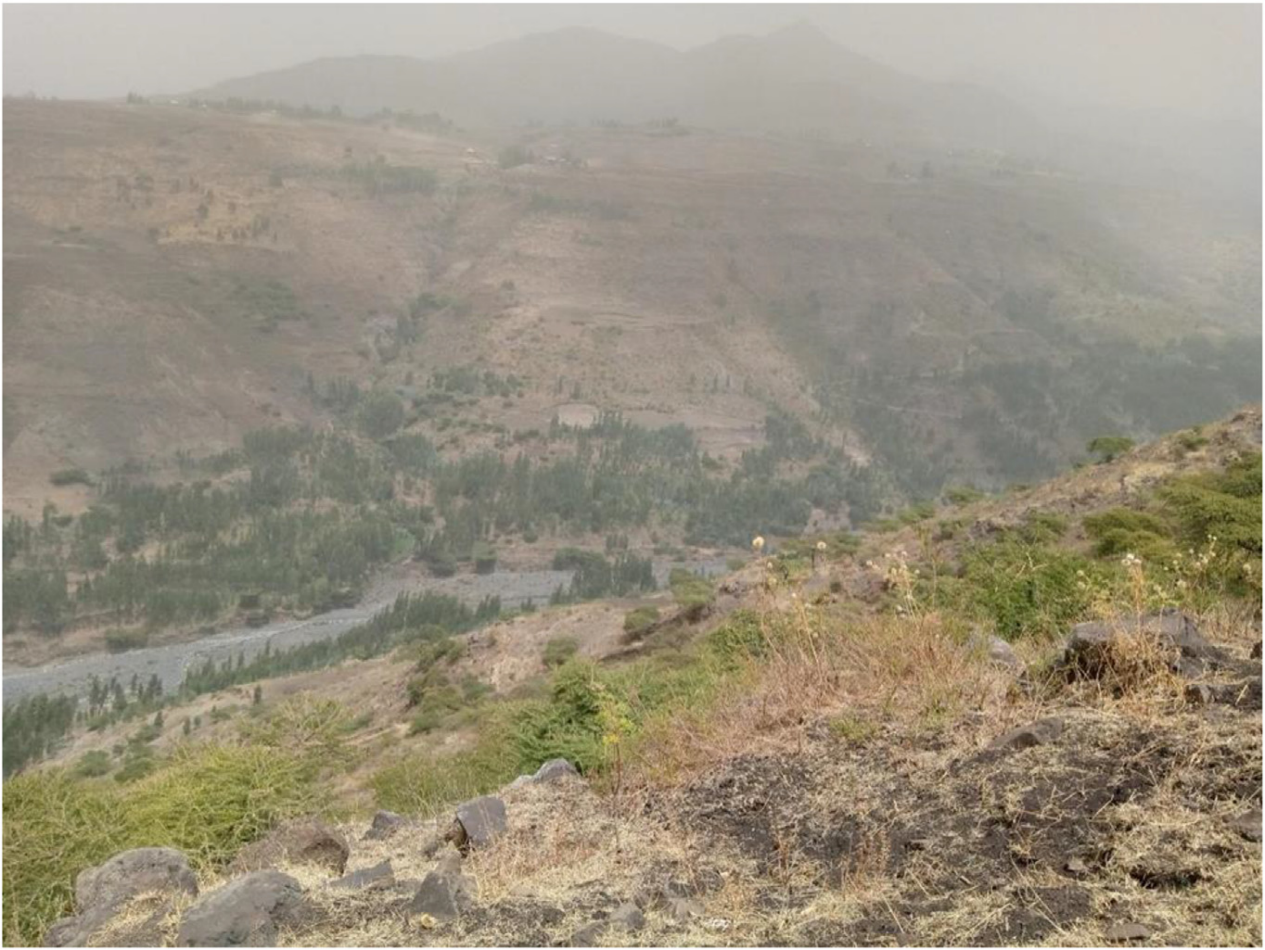
Muja greening and watershed development program started at the foot of the catchment (photo during the field observation).

Three themes emerged, guided by the research questions, namely, types of skills acquired through project-based learning, implementation of skills by project members, and how project members’ environmental attributes and livelihoods were improved through the acquisition of such skills and knowledge (Figure 2). The challenges are also highlighted.

### 4.1 Document analysis

Important information was gleaned from varied documents. There are several significant scientific documents available locally and globally. Local documentary resources included ORDA's strategic plans, project proposals, annual reports, official proclamations, established constitutions, and policy documents. Further the study used universally available EE agreements, conventions, and protocols, which generated important information on the concerns of the study.

The recent events of the African Union Summit 2020 in Addis Ababa and the Davos 2020 meetings have brought strong contributions the discussion of sustainability of the environment and development. Another meeting, the African Union Summit 2020 in Addis Abeba, advocated for the development of a sustainable Blue Economy due to its strategic importance to the implementation of Agenda 2063 flagship projects, as well as to environmental challenges and SD issues. The blue economy strategy has enabled Africa to guide the development of an inclusive and sustainable economy which supposed to contribute to continental transformation, growth, and environmental sustainability. Thus, environmental sustainability, social sustainability, and economic sustainability are the three pillars of blue economy (UNECA, 2016). The Davos 2020 World Economic Forum discussed the environmental challenges and climate changes that affect have been overwhelming the global community.

In the Ethiopian context, there is a strong and ambitious high-level government commitment to a green economy and greening agenda within the context of accelerated industrialization (Mulugeta et al., 2019). The Green Economy Strategy may be surpassed by the Green Legacy, which will be followed by a massive campaign of new plantations across the country. Non-governmental organizations have also supported the SD policy and environmental activities in different ways. ORDA's fourth revised strategic plan is strongly allied with the Africa Agenda 2063 and UN Agenda 2030. It follows that the newly developed strategic plan will be carried out in 2021. ORDA, as part of non-formal EE, has developed experience sharing, stakeholders' workshops, review meetings, and capacity building and awareness-creating training strategies (ORDA, 2017, 2019, ORDA, 2020). ORDA's evaluation, monitoring, and learning reports indicate that the organization accumulated knowledge regarding the status of ORDA's project from separate ORDA operational areas (ORDA, 2019a, ORDA, 2019b; ORDA, 2020).

### 4.2 ORDA's commitments and practical experiences

ORDA is a local NGO working to fill in the gaps of government strategy in the Amhara region. Since its establishment as a local NGO in 1991, ORDA has accumulated learning and communication strategies by evaluating its performance in the areas of environmental security and livelihoods income improvement. The performances of ORDA in EFDP are acculturated through knowledge management systems, experience sharing, information exchange and communications. This section has provided an overview of the organization's overall planning and performance to fulfill its mission of environmental security and livelihoods in the Amhara regional state. ORDA has passed through four consecutive strategic plans. ORDA was founded in 1984 in response to rural poverty as the Ethiopian Relief Organization, which was legally registered as a local NGO 1991. In contrast, the latest version, until 2020, of the mission of ORDA (2016) is to "empower poor communities and their institutions to achieve livelihoods and environmental security in the Amhara region" (P.9). Until 1993, ORDA focused on displacement and rehabilitation of the Amhara region. In this implementation period, the development strategy of ORDA is focused on emergencies, with little regard for engaged in emergencies at the cost of environmental consideration. From 1993 until 1997, ORDA exclusively engaged in development activities while environmental security was overlooked. Currently, ORDA is working on community projects.

For three decades, ORDA performed EFDP activities that have been allied with the government's environmental and development policy (EI1, EI2). The fourth revised strategic plan ends in 2020. Now, in 2021, another strategic plan will continue. One strategic plan includes a five-year time frame work for community projects. The strategic plans have been compartmentalized into each implementation year's planned activities. At the end of each implementation year, the organization prepares an annual performance report. From these reports, the organization has learns about the relevant opportunities and challenges (Table 2). The documented knowledge of the previous year is included in the preceding activities plan. Some measures are considered to address the challenges which will be documented to improve them in the next plan.

**Table 2:** Non-formal EE practices and challenges of ORDA's performance on EFDP.

Strategic plans	EFDP works of ORDA	Non-formal EE forms: skills & knowledge	Practices and challenges
I (1997–2003)	none	None	Absence of strong training; lack of short-medium-long-term training; failure to reveal policies & guidelines; unable to improve & apply implementation systems.
II (2004–8)	Empowering poor communities and being inclusive of women, landless people, unemployed young people, and people with disabilities. Participation: partnership; Environmental Rehabilitation and Agricultural & Forest Resources Development	The period is marked by massive change and footprints, enhancing the highest-highest visibility among the public, the government, donors, and partners.	Dependency syndrome; poor monitoring system, low technical knowhow of farmers; inadequate support from the community; low level of community participation & image building efforts are not to the standard.
III (2009–2013)	Natural resources & forest development: plantations; promotion of alternative energy technologies; biodiversity conservation; integrated community watershed & environmental protection; Participatory forest management; Degraded land Rehabilitation/Area closure; Promotion of fuel-efficient technologies; Gully rehabilitation.	Knowledge, skills, attitudes & practices of the local community regarding natural resources management and climate impacts have been improved.	(1) lack of adequate & efficient participation of society & other concerned parties; (2) aid-seeking attitude (reduces ownership & capacity of participation); (3) poor educational status of the people prohibits ORDA from expanding its activities; (4) absence of strong training, lack of short-medium-long term plans (5) failure to reveal policies & guidelines; (6) unable to improve & apply the implementation system; (7) not using training opportunities, conferences & workshops.
IV (2014–2020) (2016–20), Revised	Empowering poor communities and their institutions to ensure environmental security; stakeholder participation on seedling production and plantation; fuel-saving stoves and solar technology; biogas plants; watershed establishment & management; system development, and application; effective support, monitoring, evaluation, and learning; strategic partnerships (ORDA, 2021b, ORDA, 2021a)	Empowering communities & their institutions; inclusive stakeholder participation; system development, and application; monitoring, evaluation, and learning; strategic partnerships (ORDA, 2021b, ORDA, 2021a)	Documentation and sharing of good practices and organizational learning; Poor planning and reporting; difficulty of bringing behavioral change of ownership, limited capacity of staff; COVID-19 affected implementation, capacity building & program activities; a scarcity of funding (ORDA, 2021b, ORDA, 2021a).

The organization gained timely experiences with basic environmental security and community livelihoods. The environment, natural resources, and watershed management are the most visible areas or themes of intervention. In the preceding section, the pitfalls of each strategic plan in relation to non formal EE as a component of PEC are highlighted. The above-summarized challenges were categorized into participation, attitudes, poor educational status, insufficient training, policy, limited capacity building, image building, monitoring, and evaluation. Besides, the details of the above results could be described below.

#### 4.2.1 First strategic plan

The first strategic plan of ORDA was developed for the organization, which was implemented in the years 1997–2003 (ORDA, 2009, ORDA, 2009a, ORDA, 2009c). The plan focused on natural resource protection, agricultural development, rural water supply, rural feeder road construction, resettlement, and rehabilitation of migrants, emergency food aid, and food store construction. ORDA has since then considered and integrated environmental issues into the development process. This was done to comply with a government policy centered on environmental protection and rehabilitation. After its implementation, it was reported that there was a low commitment to the staff, a high turnover of staff, and a problem with addressing the turnover of staff. The document analysis findings show that the strategic plan document was not strictly followed and applied. It was preoccupied with humanitarian food aid, and, as a result, natural resource protection was inadequate. Moreover, there was no non-formal EE approach used as a major PEC tool to empower the stakeholders in that context. It also demonstrates that there was little community involvement during the project's implementation. These were the major implementation strategic factors that hindered the ORDA's EFDP in the Amhara regional state. This was the first, small step toward improving the region's environmental security.

#### 4.2.2 The second strategic plan

The focus of the second strategic plan (2004–08) was in place after evaluating the performance of the first strategic plan. During its second strategic plan and in its implementation years 2004-8, the organization (ORDA, 2016) identified priorities in agricultural development and environmental protection, forest resource development, water resources development, capacity building, and community development, disaster prevention, and relief programs. In addition, the strategic plan for the EFDP issues got much more attention than the first strategic plan of ORDA. It is also characterized by enormous change and footprint, enhancing the highest visibility among the stakeholders (public, government, donors, and partners). However, challenges are manifested as inadequate and inefficient participation of the community and other stakeholders. Besides, the aid-seeking attitude of the project community members reduced ownership and capability of genuine participation. The poor educational status of the community prevented the organization from expanding its activities.

#### 4.2.3 The third strategic plan

The key focus of the third strategic plan (2009–13) was carefully followed after favorable evaluation of the outstanding performance of the second strategic plan. The third strategic plan of the local organization was typically implemented in the successful years of 2009–2013 (ORDA, 2009, ORDA, 2009a, ORDA, 2009c). It also had reduced and focused its priorities on natural resources development, water resource development, food security, and agricultural programs. Its accomplished mission was to enhance the sustainable livelihoods of the local people and ensure environmental security in the region. ORDA's third strategic plan performance reports indicated that empowerment and meaningful participation were sufficiently emphasized in the implementation strategy (ORDA, 2014). In this strategic plan, non-formal EE has received proper attention and was used as an effective instrument to empower and enable participation in the community projects.

Non-formal EE, therefore, has become one of the strategic community empowerment instruments of the organization. It helped the community projects as training of trainers on Adult Learning Principles and Communication, which could be provided to district experts, project community members, and project staff (ORDA, 2014). Another dimension of non-formal EE its relevance as a learning forum for scaling up purposes (ORDA, 2012). For instance, a learning forum was established in April 2012 with the key objective of scanning ORDA's "development approaches and perceiving the intriguing possibilities of scaling up" (ORDA, 2014, p.72). However, the official report has been criticized for its lack of active participation from members of the target community project. Moreover, low project community participation has been reported. ORDA's lifelong learning and scaling up strategy, which includes capturing knowledge and experience of opportunities and obstacles encountered during project execution using ORDA's learning forum, is a great asset.

ORDA firmly believes that knowledge management can improve the generation of information and data capture, proper storage, dissemination and scaling up. Other point to make note of in the third strategic plan is ORDA's participation of local women, particularly in the learning process and dissemination of best practices are another valuable asset. Learning forums were essential to institutionalizing the strategy, helping more to achieve the vision of ORDA.

However, ORDA has learned from many challenges. A low level of knowledge management and learning could be the input for further improvement in the ORDA's strategic thinking and dynamism. There was also a low level of a strategic role in improving the capacities of the regional project community members in the scaling up of organizational capacities in project design, planning, monitoring, and evaluation. Moreover, the monitoring and evaluation results were seriously unconsidered for learning and as a management tool for further improvement and strategic implementation as one of the learning strategies of the organization.

#### 4.2.4 The revised fourth strategic plan

The fourth strategic plan has been revised to align ORDA's program with the government's Growth and Transformation Plan II (GTP-II). The fourth strategic plan of the organization (2014–2018) was first implemented in 2014–15, before a revised plan was presented (2016–2020) to meet the revised Ethiopian government development policy. ORDA's strategic plan gives due attention to its mission to work to improve the environmental security of the region. It has shifted to a unique scientific approach that focuses on the Theory of Change (ORDA, 2016; Rogers, 2014). It has been adapted to ORDA's key aspects of thinking, behaviors, and possible outcomes (ORDA, 2016).

The revised fourth strategic plan has addressed the issues of the SD Goals. One of the goals is Goal-13, which calls for immediate action to combat climate change and its consequences. The other goal is Goal-15, which aims to protect, restore, and promote sustainable use of terrestrial ecosystems, sustainably manage forests, combat desertification, and halt and reverse land degradation and biodiversity loss. Goals 13 and 15 are thus directly aligned with the vision and mission of ORDA. Further, the fourth strategic plan is consistently allied with national and international agendas. Currently, ORDA's program consists of different programs divided into the following categories: environment and forest development, water and irrigation programs, agriculture and disaster risk management programs, youth enterprise and private-sector development, and gender unit. The current study has focused on the sub-divisions of five of the EFDPs of ORDA. The program is aimed at establishing environmental security and livelihoods of the community. The EFDP is also sub-divided into forest development, biodiversity, watershed development, soil, and water conservation, and climate resilience. The research investigates the project activities of five programs. Although the theory of change is used as one of the frameworks to implement the EFDP, it has not yet received much attention.

ORDA's non-formal EE activities include training of stakeholders on forestry-agroforestry, biodiversity conservation, alternative technology, watershed management, experience sharing on natural resource development, workshops-biodiversity conservation, strategic partnerships for mutual benefits, improved management information systems, stakeholders' relationships, improved monitoring, evaluation, and learning systems through executive and deputy directors, communication and IT, and other programs and services (ORDA, 2021a, ORDA, 2021b). Capacity development activities have increased project beneficiaries' experience, knowledge and skills in the environment, forest development and climate change adaptation. These educational activities are primarily concerned with technical support.

The attitudinal challenges of the community projects are obstacles to start implementations. The community has resisted the launch of the community projects before the start of the actual implementations. Second, the limited knowledgeable of community and experts to develop a sense of ownership is challenging during the implementation of project interventions. It is clearly a practical constraint of non-formal EE. Third, there is a fundamental lack of potential inclusion of young people and local people with disabilities. Fourth, ORDA documenting and sharing best practices and organizational learning (ORDA, 2021a, ORDA, 2021b). Fifth, the pandemic has made it impossible train experts and the community full face-to-face interaction. In rare cases, few funding partners deactivate project implementation. As a result, a lack of funding for the EFDP has limited non-formal EE capacity building.

### 4.3 Interview, focus discussions and observation

Data was collected from the Enfiranz, Derita, Muja and Eyella project community and analyzed thematically. Thematic analysis is developed on the components of EE indicated in conventions, agreements, and various academic discourses that include B-MASKA. The study has identified EE in terms of training, experience sharing, demonstrations, forums, and field visits. In essence, EE goes beyond awareness, knowledge, attitude, skills and participation. It is linked to sensitivity, understanding, motivation, solutions and resolution. It goes to decision-making and taking actions to respond to environmental challenges (Rahman, 2011). It could be considered as Cox's (2010) pragmatic function of EE. Therefore, this study has considered human sources to investigate how ORDA has implemented community projects and achieved the desired outcomes. Data collection techniques included in-depth interviews, focus group discussions and observation. Observation as a data collection technique was used to obtain data from the seedlings, nurseries, watersheds, and greening of the Enfiranz, Derita, Muja and Eyella project community. Agro-forestry sites which the Derita, Muja and Eyella project community adopted for their gardens were observed.

The findings emerged from the six focus group discussions and seven individual in-depth interviews held at the three study sites. Moreover, three experts’ in-depth interviews were conducted. During these in-depth interviews, participants were invited to ask open-ended questions about the opportunities, challenges, and impacts of non-formal EE on helping them to deal with environmental challenges and livelihood problems.

Environmental education is a strategic tool to create awareness and exchange information. It also prepares environment and livelihood knowledge and skills for the SD and well-being of human survival (Mohammed et al., 2006). The capacity building of ORDA in the community projects has been provided to numerous targeted participants who are allowed to support the desired outcome of the community projects. The training is offered to diverse stakeholders, such as project community, experts, and extension workers. The training is provided to empower and boost confidence, knowledge, skills, experience, and attitudes. These include technical skills, livelihood skills development, biodiversity conservation skills, knowledge management skills, and watershed development skills. Moreover, entrepreneur skills have been provided for young people and women. Livelihood income techniques have been developed over a considerable time.

The training on agroforestry is provided to the above-mentioned (Table 3) training participants. Training represents an eye-opening strategy in rural society. Non-formal EE establishes and maintains coexistence between the environment, nature, and human beings. An expert has been (EI3) prepared for various activities. It is stated that, "we are often trained in integrated watershed development, soil and water conservation, and participatory forest development and management strategies" (EI3). The project community at Eyella intervention sites has confirmed their participation in ORDA's workshop held at Bahir Dar (EI6). However, the majority of participants in the project community obtained no training (CI1, CI2, CI3, CI4, CI5).

**Table 3:** Experiences of ORDA's capacity building of staffs and stakeholders.

Participants	Themes	Descriptions	Source
Community	Agroforestry, Watershed, Technology Climate change	Capacity development training was provided to targeted community members. Moreover, training was provided on forestry and agro forestry development…integrated watershed management...fuel saving stove technology … climate changes adaptation.	ORDA, 2019b, ORDA, 2019a, p.9
Government staff	Agroforestry, Watershed, Energy, Climate change	The capacity development training was provided to government staff on agroforestry, integrated watershed management, fuel saving energy and climate change adaptation.	ORDA, 2019b, ORDA, 2019a, p.9
ORDA's staff	Agroforestry Knowledge, Skills	ORDA has been working to develop the technical knowledge and skills of its expertise. In this regard, training and experience exchange were organized for staff on agroforestry	ORDA, 2019b, ORDA, 2019a, p.9

Another expert from Libokemkem district explained that ORDA had various capacity-building methods. He mentioned training as a priority area. ORDA has provided a day of discussions and practical workshops on "how to apply recent research findings, their usefulness, their application, and their handling" (EI3). He further indicated that another form of capacity building is provided through an exchange of experiences (EI3). However, he added that mutual learning had progressed through working together. Collaboration as "another skills development strategy remains to be a practical training opportunity that allows people to acquire real knowledge by collaboratively working and inter-personally sharing knowledge and skills " (EI3).

ORDA, further improves the capacity of stakeholders through non-formal EE. In this form of diversity, it has impacted short-term and long-term education and training (EI1, EI2) conducted in collaboration with agriculture offices and provided at the administrative and district levels (EI1). The second expert (EI2) has mentioned that training is given to surveying community members. The basin committee has been trained. However, the expert expressed that all project community members could not receive the training (EI2). He asserted that "it is difficult to provide training for all in the community." The training requires target trainees based on capacity limitations. He mentioned how they selected them; "We identify and prepare those who are weak. In the case of soil and water conservation, it is frequently based on awareness creation and supervision "(EI1). He further mentioned technical support could be given to the soil and water conservation participants of the project community (EI1). However, in the community project, ORDA uses an inquiry-based strategy that receives a need-based request. "They say we were lacking capacity. They say we have a capacity gap now. They say that, according to the training we received from ORDA, we can repair or correct damaged work. They repeatedly ask ORDA for training " (EI).

General information is provided by an expert regarding the limitations of the training offered to community (EI2). Short-term training is constrained due to lack of resources. The resources from donors are limited to the project work but with insufficient attention to capacity building. They would rather focus on activities or implementation of community projects with less emphasis on capacity building. The expert asserted this limitation as follows:

The training is limited. Trainers are additionally required to inform professionals below. Training is associated with resources. ORDA does not pay attention to the training because there is lack of funds. Since no organization works without a government structure, we provide most of the training to government employees. There is no appropriate training for professionals. A few things make us better. There are very few training based on strategy. Most are donors. There is no money for training. There are opportunities for long-term education. But the standard is not good. There are limitations. The parameters are completely different from one program to another. That's what the organization remarks. Education additionally provides quota-based opportunities. The training focuses on the political supporter rather than the professional (EI2).

Another expert from ORDA explains that ORDA had different capacity–building methods. He mentions training as a priority area. ORDA provides a day of discussions and hands-on workshops on "how to apply new research findings, their usefulness, their application, and their handling" (EI3). He further indicates that other forms of capacity building are provided through an exchange of experience (EI3). "Experiences are often seen and applied here. The community asks us to bring experience from other ORDA implemented projects to other sites. We show them sites and they can visit them. We want our community to study the area with better experience and visit it earlier "(EI3). They confirm that mutual learning benefits them through working together. Collaboration is a possible means of "practical training that allows people to possess the exact knowledge by working together in practice" (EI3).

In conclusion, non-formal educational training is provided in three environmental lessons (EI1, EI2, EI3). Being in the area where the farmer works is working together. Collaborative or mutual learning is most favorable. It is argued that in a collectivist culture, this kind of learning has a strong influence on acquiring knowledge and skills. It can also build and strengthen collaborative learning. This one is a kind of experiential learning. Learning by doing and engaging in the experience could be crucial to managing community projects. The second strategy is by providing program training. It could be face-to-face discussions and workshops. The third is by sharing and exchanging experiences. It followed mutual learning.

#### 4.3.1 Benefits of acquired knowledge and skills

##### 4.3.1.1 How did the skills and knowledge acquired help the community to deal with the environmental challenges they experienced?

High intensity flooding challenged environmental rehabilitation, restoration and greening. To avoid these challenges, the project community acquired knowledge and skills through various capacity building mechanisms on watershed management and bench terracing. Participant EI1 said: "we identify and train those who are weak. Besides, in the case of soil and water conservation, it is often based on awareness creation and supervision". Technical support was provided. In fact, one of the objectives and components of non-formal EE is to acquire knowledge (Athman and Monroe, 2001). This occurs through a mutual learning process of participating in community project activities. The project community could be supervised and corrected while they were working in the fields (CI5, CI6). Participation in experience sharing and training at all levels could be one of the best opportunities to acquire environmental knowledge and skills. The study indicates that project community members acquire knowledge through a variety of experiences and information sharing opportunities. They also get a basic understanding of the environment and its related problems. The knowledge gained during the training workshops, forums, and experience sharing and field visits empowered project community members to invent new ways of greening, watershed development, and farming. The project community members are also able to have versatile communication about their interaction with the environment and among their members. With the help of the ORDA facilitator, project community members managed to secure and sustain a bench tracing, establish an indigenous association, protect the communal land, and a watershed committee to conserve the environment and biodiversity for livelihood development, forest greening, and farming. Besides, the local indigenous knowledge of the project community members is used to manage challenges associated with greening and watershed management. Knowledge acquired during the field visits, dialogues, and training enabled them to come up with an effective mechanism for dealing with environmental challenges in their surroundings (Figure 2).

The project community members have performed bench terracing as the best approach to cope with hillside watershed development. It helps them to perform forest and greening programs. A participant said that" The applied technology improved crop production (EI1). Moreover, another participant stated that “In our village water is very scarce and we could not cultivate enough crops for our families. We thank the NGO for the borehole and the workshops which enabled us to develop ways to irrigate our crops"(CI7). The workshop also taught the project members how to enrich the less arable land. Most of the project members alleged they used their skills and knowledge to open small businesses. Participant (CI7) states: "the business skills I acquired helped me to open some business outlets even outside the community”.

##### 4.3.1.2 How did the acquisition of skills and knowledge and subsequent environmental and forest development activities change the livelihoods of households?

Non-formal EE enables the project community members to become more productive and effective citizens through livelihood skills development. The projects of ORDA are aimed at increasing the productivity of farmers and improving their livelihoods. The objective is to restore the degraded forest, improve groundwater recharge rate, and protect downslope dwellers and their farmlands from flood damage by reducing soil erosion. Moreover, beekeeping is a means of income generating entrepreneurial skills for both the young and the farmer.

Findings indicated that the project community members were engaged in greening and watershed programs which earned them income to fend for their families. The knowledge acquired during project-based learning was transferred to community members who had not attended the workshops. Some members confessed that they produced more than enough for their personal use, and thus shared it with those who were lacking. Empowerment expanded to other community sites. The development of gardens was rolled out in other villages, which means that the knowledge the initial participants acquired was spread to others outside of Enfiranz, Derita, Muja and Eyella villages.

The empirical findings also shed light on the considerable importance of non-formal EE, whereby the prominent community members themselves come up with practical suggestions and take the lead in addressing their fundamental problems. The comprehensive study affirmed the importance of community members taking the initiative to solve problems (Saribas et al., 2017).The action, also referred to as project-based learning (Genc, 2015), developed positive attitudes towards project community members towards improving their livelihoods. Through participatory and collaborative learning, the project community members developed competencies related to essential teamwork, communication, self-management, critical thinking, and problem solving, which are key for anyone wishing to compete in the global world. The project community members demonstrated such skills, e.g., they collaborated well as a team and shared opinions in allowing decisions about what could work for their project. Unlike government-initiated programs that dictate what people must do to improve their lives, the strategies used by NGO promote ownership of actions, hence they are successful.

Improved skills and practical knowledge have led to improved environmental security and decent livelihoods. The recent EFDP methods acquired were extended to project community members' homes’ where backyard gardens were developed. Key participants sufficiently indicated how the backyard gardens invariably led to improved livelihoods of project community members and local people in their neighborhood. The strategies they embarked on to respond to environmental challenges led to co-benefits which resulted in increasing environmental security and improved livelihoods (Newell et al., 2018). Key findings also indicate that the increased crop production and forest harvesting earned project community members and the community at large income to fend for their families and to cover other expenses. The local youth and the community gained entrepreneurial skills and knowledge to operate small businesses selling firewood for housing purposes, and harvesting honey. Consistent findings also indicate that extending the EFDP to social institutions like schools and clinics not merely increased the number of business outlets for the project team, but it also promoted healthy living. The selling of forests to community members improved the diet and prevented the community from acquiring diseases borne from not consuming a proper diet.

#### 4.3.2 Opportunities and challenges

The result indicates that several opportunities have been found to move the organization and the community forward. Meanwhile, implementation and sustainability challenges are documented and witnessed by the organization and community respectively. However, correcting the challenges has been overlooked and rolled back in several implementation years.

##### 4.3.2.1 Opportunities

**Figure 3:**
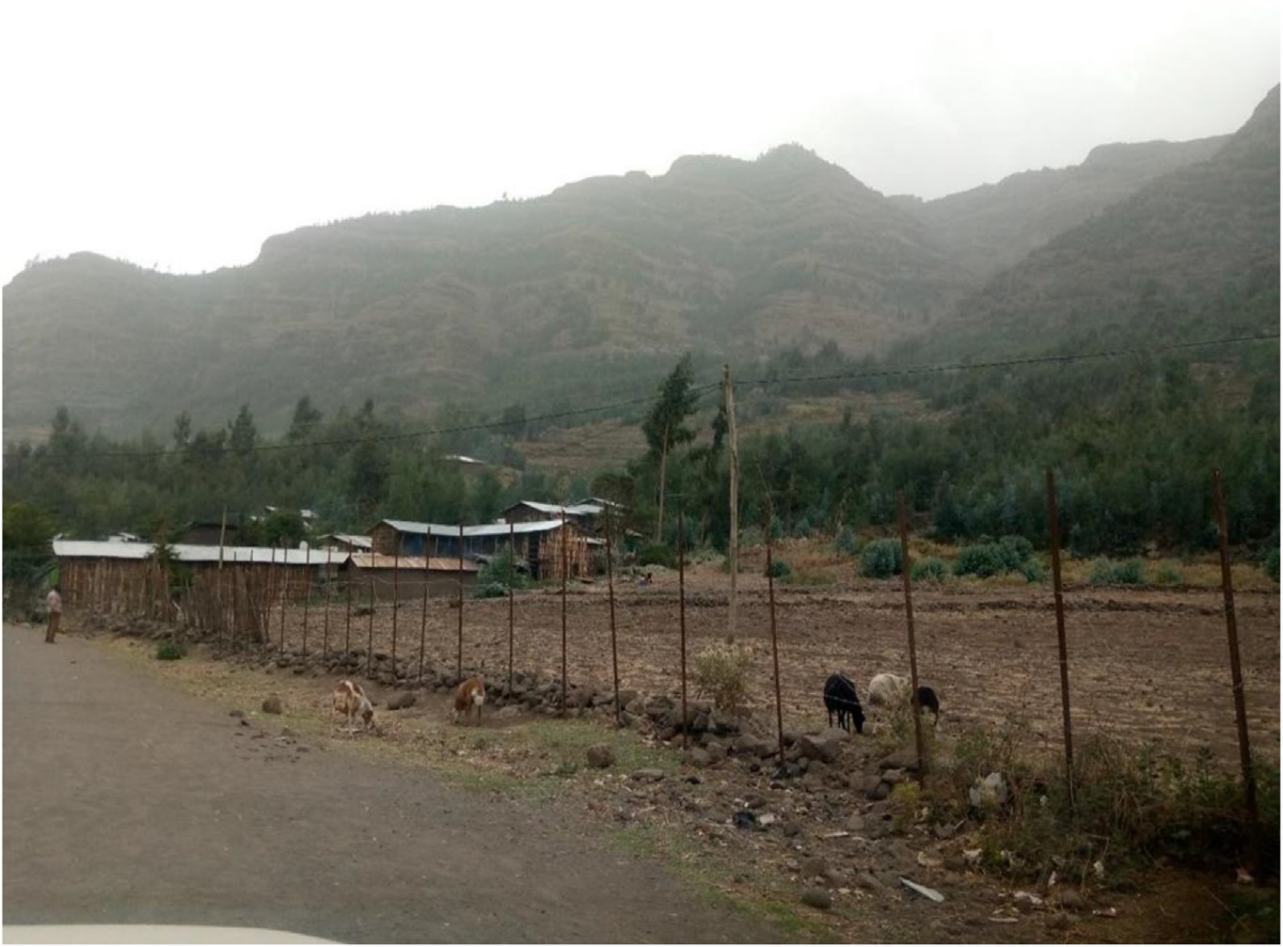
Eyella greening and watershed development program started at the foot of the catchment (photo during the field observation).

The growing focus on addressing environmental issues, livelihood challenges, and other pertinent issues by local, national and international communities remains to be a golden opportunity for ORDA's EFDP. The recent phenomenon of the Green Legacy, a continuing campaign across the nation, could be marked as an environmental movement that has motivated all the citizenry for two years and is believed to have a sustainable impact. It is supposed to continue. It would also be assisted by environmental education, as well as key stakeholders' meaningful participation and effective advocacy. Moreover, ORDA has emphasized the importance of local government policies and successful strategies used to achieve SD, such as agricultural development-led industrialization, rural and agricultural development policies, industrial development policies, environmental policies, climate-resilient green economy, charities and social agency proclamations, youth and women's policies, and so on. The coexistence of various communities based on society's institutions (edir, watershed committee, forest committee, development army, etc.) has been identified as a community opportunity. Gradual acceptance of ORDA by potential donors, communities, and legitimate government structures presents an important opportunity for the practical implementation of community projects among the rural Amhara people. ORDA has contacted the community and started a dialogue with them through the government structure. The availability of communal lands is crucial (Figure 3).

The prolonged problems of flooding, frequent drought and soil infertility have been some of the possible opportunities for intervention, motivation, information exchange, mutual learning, experience exchange, field visits, training, cooperation and participation. Participating youth and women in the learning process and dissemination of information represent valuable opportunities for the project community.

##### 4.3.2.2 Challenges

The dynamism of the nonprofit organization's performance continued. The coded and tacit knowledge of the community project manifests in the project's life cycle and beyond. As stated above, there are several documented challenges experienced by ORDA during the life cycle of the projects. ORDA's intervention implementation strategies intentionally include inclusive community participation; effective communication; explicit image building; successful partnership and fruitful collaboration; the Integrated Watershed Management approach; and knowledge management. However, limited capacity-building support to staff and key stakeholders; low monitoring, evaluation, and learning and database management; limitations in community participation, and deep-rooted dependency syndrome are the major challenges manifested before, during, and after the practical implementation of community projects. It has been noted that knowledge management remains the process of capturing, distributing, and effectively using knowledge. ORDA believes knowledge management is a basic tool to build the implementation capacity of the staff, the target communities, and government stakeholders to ensure project or program success and sustainability. However, there are several non-formal EE challenges as adequate training was limited due to the availability of resources and funds. Also, the majority of the training is provided to government employees (EI2). The expert asserts that "there is no specialized training for professionals".

## 5 Summary and conclusion

### 5.1 Summary

ORDA uses the term stakeholders' participation in representing the community, government staff, and donors. Stakeholders are the public who participated in the EFDP of ORDA since the organization started strategic planning development and implementation. The organizational documents assessed for this study purpose are scientific project papers, four strategic planning documents, and their respective annual performance reports. The donors’ participation in contributions and financial releases has been dependent on the annual performance report. Besides, the organization develops insights for knowledge management for further implementation strategies. The major focus areas during the course of action were to empower poor communities and their institutions aimed at ensuring environmental security. The stakeholders received knowledge and skills for capacity building activities such as forestry/agroforestry, biodiversity conservation, alternative energy, and watershed management through experience sharing, pieces of training, and workshops (ORDA, 2019b, ORDA, 2019a, 2018, 2017, 2016 & 2014). Findings indicate that most of the project community members were illiterate, and prior to engaging them in project activities, they needed to learn basic knowledge and skills. More community members were attracted to being part of the project because of the mobilization and awareness creation campaign provided by ORDA and the community leaders. Initially, there was resistance to accepting the community projects. Through various discussions, they agreed to proceed to participate in its implementation. However, several challenges have been road blocks to the implementation of the community projects.

### 5.2 Conclusion

The study focused on a relatively less explored area of how non-formal EE in the form of project-based learning was used to capacitate and empower Enfiranz, Derita, Muja and Eyella communities which practiced EFDP greening and watershed management with acquired skills and knowledge needed to improve environmental quality and forest production. These conclusions are derived from both document and thematic analysis.

The scientific document analysis indicates that ORDA's comprehensive EE strategy since its official establishment in 1993 could be manifested in many ways. These include training, learning forums used for scaling up, documenting opportunities, challenges, and knowledge management, and institutionalizing the strategy used for scaling-up attainments, empowerment of the community, and knowledge management. These could be considered as inputs for developing other plans. Thus, ORDA has used non-formal environmental strategies: (1) Adult Learning Principles and Communication; (2) Stakeholder Participation Theory; (3) Practical Knowledge Management and (4) Theory of Change.

However, the overall investigation of this study shows that a low level of knowledge management and lack of awareness are important limitations. There is also a low level of strategic role in enhancing the capacities of the regional project community members in the scaling up of organizational capacities in project design, planning, monitoring, and evaluation. Monitoring and evaluation results are not adequately considered for learning as well as a management tool for further improvement. These tools are not integrated into the learning, participatory, and legitimacy strategies of the organization's project community. The ORDA worked in an unfavorable Ethiopian government's political and development policy context. It has no full-fledged freedom to consider all walks of life in all society. It has been bound in government contexts. The context of the organization has a little bit of a partially open system. The dynamic nature of the organization system is signified and well manifested, but bounded by government systems and respected policies.

The thematic analysis reveals that ORDA is more than the facilitators of such training, which culminated in the improved environmental quality of the project community in the three above-mentioned zones. Greening campaigns, the acquisition of entrepreneurial skills, and skills and knowledge to deal with less arable land and highly degraded and eroded land were found to be essential in sustaining the lives of those communities that launched greening programs. The use of excreta to develop manure and irrigation systems to solve the problem of drought appeared to be one of the strategies to prepare the soil with a low PH for a high crop yield. Evidence was provided to suggest that there are benefits to community projects aimed at enhancing learning, which aligns with the theory of project-based learning. We propose project-based learning be used as a tool for community empowerment initiatives aimed at responding to environmental problems. No study was examine to investigate NGOs that supported community members' initiatives to address environmental challenges, to fill the gap in government attention to climate change problems. We believe this study could contribute to the body of knowledge on strategies to mitigate environmental challenges confronting communities that practice environment and forest development programs. The study also produced evidence of the importance of greening and watershed development programs for out-of-school youths and adults in acquiring fundamental skills and knowledge to improve livelihoods.

## Declarations

### Author contribution statement

Mekonnen Hailemariam Zikargae; Amanuel Gebru Woldaregay; Terje Skjerdal: Conceived and designed the experiments; Performed the experiments; Analyzed and interpreted the data; Contributed reagents, materials, analysis tools or data; Wrote the paper.

### Funding statement

This work was supported by the Faculty of Humanities for Research, Postgraduate and Community Services, 10.13039/501100005872Bahir Dar University.

### Data availability statement

Data will be made available on request.

### Declaration of interests statement

The authors declare no conflict of interest.

### Additional information

No additional information is available for this paper.
